# Differentiation of single lymphoma primary cells and normal B-cells based on their adhesion to mesenchymal stromal cells in optical tweezers

**DOI:** 10.1038/s41598-019-46086-y

**Published:** 2019-07-08

**Authors:** Kamila Duś-Szachniewicz, Sławomir Drobczyński, Marta Woźniak, Krzysztof Zduniak, Katarzyna Ostasiewicz, Piotr Ziółkowski, Aleksandra K. Korzeniewska, Anil K. Agrawal, Paweł Kołodziej, Kinga Walaszek, Zbigniew Bystydzieński, Grzegorz Rymkiewicz

**Affiliations:** 1 Department of Pathology, Wrocław Medical University, Marcinkowskiego 1, 50-368 Wrocław, Poland; 2 Department of Optics and Photonics, Wrocław University of Science and Technology, Faculty of Fundamental Problems of Technology, Wybrzeże Wyspiańskiego 27, 50-370 Wrocław, Poland; 3 Department of Statistics, Wrocław University of Economics, Komandorska 118/120, 53-345 Wrocław, Poland; 4 2nd Department of General and Oncological Surgery, Wrocław Medical University, Borowska 213, 50-556 Wrocław, Poland; 5 Division of Pathology, Sokołowski Hospital Wałbrzych, Sokołowskiego 4, 58-309 Wałbrzych, Poland; 6 Flow Cytometry Laboratory, Department of Pathology and Laboratory Diagnostics, Maria Sklodowska-Curie Institute-Oncology Centre, Wilhelma Konrada Roentgena 5, 02-781 Warsaw, Poland

**Keywords:** B-cell lymphoma, Optical manipulation and tweezers

## Abstract

We have adapted a non-invasive method based on optical tweezers technology to differentiate between the normal B-cells and the B-cell non-Hodgkin lymphoma (B-NHL) cells derived from clinical samples. Our approach bases on the nascent adhesion between an individual B-cell and a mesenchymal stromal cell. In this study, a single B-cell was trapped and optically seeded on a mesenchymal stromal cell and kept in a direct contact with it until a stable connection between the cells was formed in time scale. This approach allowed us to avoid the introduction of any exogenous beads or chemicals into the experimental setup which would have affected the cell-to-cell adhesion. Here, we have provided new evidence that aberrant adhesive properties found in transformed B-cells are related to malignant neoplasia. We have demonstrated that the mean time required for establishing adhesive interactions between an individual normal B-cell and a mesenchymal stromal cell was 26.7 ± 16.6 s, while for lymphoma cell it was 208.8 ± 102.3 s, p < 0.001. The contact time for adhesion to occur ranged from 5 to 90 s and from 60 to 480 s for normal B-cells and lymphoma cells, respectively. This method for optically controlled cell-to-cell adhesion in time scale is beneficial to the successful differentiation of pathological cells from normal B-cells within the fine needle aspiration biopsy of a clinical sample. Additionally, variations in time-dependent adhesion among subtypes of B-NHL, established here by the optical trapping, confirm earlier results pertaining to cell heterogeneity.

## Introduction

Non-Hodgkin lymphoma (NHL) cells tend to home in bone marrow (BM), indicating that this microenvironment is favourable for lymphoma growth and proliferation^1,2^. The incidence of BM involvement strictly depends on the lymphoma type and stage, reaching up to 80% in mantle cell lymphoma^3–5^. Importantly, the biology of NHLs is largely influenced by the mesenchymal stromal cells (MSCs) that are critical in lymphoma’s progression^6^ and resistance to chemotherapy^7^. It is well established that MSCs have immunoregulatory abilities and can support B-cell viability^8–10^.

Cells interact with their microenvironment what triggers several signalling processes, including survival, differentiation, and migration^11,12^. The basic knowledge of physical and functional characteristics of individual cell-to-cell and cell-microenvironment interactions is essential for understanding the unique cells’ behaviour in health and disease. However, traditional adhesion and transwell assays extract an average amount of information from a large number of cells, but do not allow obtaining data at a single-cell level. Thus, a variety of techniques have been successfully adapted to characterise these processes at a single-cell level, including atomic force microscopy (AFM)^13–15^, micropipette aspiration^16,17^, and, finally, optical tweezers (OT)^18–22^.

To measure cell-cell interaction using an AFM, a cell is attached to a cantilever tip and the deflection of the tip is monitored as the cell is brought into contact with a neighbouring cell^23^. However, the probe fabrication techniques reduce the stiffness range that is achievable, resulting in stiff cantilevers^24^. Hence, the stiffness and applied force cannot be spontaneously and flexibly changed during measurements, what can easily damage the biological membranes^25^. In another technique called micropipette aspiration, the micropipette is positioned perpendicular to the surface of the adherent cell and a constant-rate aspiration pressure is applied^26^. The limitation of this method in relation to the cell-cell interaction study is primarily the need for protein scaffolding of the cells to avoid membrane rupture when they are pulled by a nanoneedle^27^ and inherently very low throughput (5–10 cells per day).

In this work, we have used the optical tweezers that are capable of sterile, minimally invasive trapping and moving the cells without mechanical contact. The appearance of OT and derivative technology in the late 1980s offered contactless, precise control over regulation of single-cell adhesion that has been demonstrated *in vitro*^28,29^ and *in vivo*^30–32^ in numerous works. Apart from mammalian cells, OT have also been applied in a range of bacterial^33–35^ and fungal^36^ cell adhesion studies.

The inventor of OT, Artur Ashkin^37–39^, was awarded the with one half of the Nobel Prize in Physics 2018 “for the optical tweezers and their application to biological systems”^40^. This achievement greatly highlights the major impact of this method on the uncovering of fundamental aspects of molecular and cellular biology. Nowadays, cell manipulation is one of the most impactful applications of optical tweezers and it has a number of advantages over the other single- cell adhesion assays. First, using an optical approach eliminates the mechanical contact with the cells and localised membrane stretching, which is required in AFM or micro-pipette techniques. That removes the risk of damage to soft cell membranes and interference in the normal shape and functions of the cell^41,42^. Second, OT are a unique technique covering the lower range of forces operating at 0.1–100 pN compared to ~5–10,000 pN of the AFM^43^. Thus, OT have a higher sensitivity for detection of small forces and are, therefore, more appropriate for local viscoelastic studies of the cell membrane. Additionally, the right choice of laser power and wavelength can minimise photodamage to living cell which is an important limitation during long time analyses^38^. Third, OT allow for simultaneously following the manipulations induced by multiple probes at different sites of the cell^41^.

To the best of our knowledge, optical tweezers have not been previously applied to the normal and lymphoma cells differentiation in time scale based on cell-cell adhesion that we have reported here. However, the optical setup used for malignant-normal cell differentiation has been already described in numerous studies. The first attempt was of Guck *et al*. who used microfluidic optical stretcher to monitor the changes in the cell elasticity during the progression of human breast epithelial cells from a normal to cancerous and metastatic state^44^. In turn, Chan *et al*. were the first who differentiated transformed Jurkat (T-cell acute lymphoblastic leukemia) and Raji (Burkitt’s lymphoma) cell lines from their normal counterparts using the confocal Raman spectroscopy in combination with optical trapping (laser tweezers Raman spectroscopy, LTRS)^45^. They demonstrated that single-cell Raman spectra provide a highly reproducible biomolecular fingerprint of each cell type. Next, Zheng *et al*. have adapted a similar technique to differentiate patient-derived colorectal cancer cells from normal epithelial colon cells^46^.

Referring to our experimental model based on lymphocyte B- stromal cells adhesion, few previous studies have applied optical trapping to successful unravelling of the initial interactions between both healthy and abnormal blood cells and their microenvironment. Early work establishing the initial stages of homing the hematopoietic stem cell (HSC) to the bone marrow stroma was published in 2002 by Askenasy and Farkas^47^. Importantly, authors developed *in vivo* assay for the assessment of cellular adhesion and viability *in situ* in physiological conditions. In 2013, Hu *et al*^48^. and Gou *et al*^20^. published works describing leukemia- stromal cell interaction at a single-cell level through manipulating cell adhesions with optical tweezers. A little later, a similar model (i.e., leukemia-stromal cell interactions in OT) was used to determine the influence of SDF-1/CXCR4 signalling pathway on cell adhesion and migration^19^. Gou *et al*. verified using optical tweezers that blocking the signalling pathway of SDF-1/CXCR4 through drug treatment (i.e., AMD3100) significantly affected the adhesion ability of leukemia cells to stromal cells. Optical trapping in this work also enabled studying the initial cell adhesion and cell migration at different contact sites by precisely assembling a leukemia cell at varied distances from the nucleus of the stromal cell. Recently, the optical trap was used to directly control the initial adhesion of a dendritic cell and a T-cell and, subsequently to terminate the interaction at a defined point in time^49^. By determining a relative interaction force, the authors were able to confirm the role of a specific antigen (-OVA group) in the initial cell-cell interaction at a single-cell level. Importantly, in this study, single T-cells were optically trapped with no exogenous beads added to the system, as we did in our research.

Hitherto, optical trapping experiments on cell-to-cell adhesion conducted in time scale have been presented first by Landiwala *et al*.^21^. In their *proof of concept* work, they provided strong evidence that individual neural stem cell (NSCs) adhesion dynamics proceeds in time scale of only a few seconds. Similar approaches, quantifying the differences in the time taken for cell-to-cell adhesion to form, enabled assessment of the differentiation status of two neural tumour cell lines: the human neuroblastoma SK-N-SH and rat C6 glioma cells^22^. This work showed for the first time that the minimum mean time for adhesion to occur significantly increases for malignant cells of both cell lines compared to normal NSCs. In addition, authors successfully implemented the above approach to detect minute changes in cell-to-cell adhesion provoked by a drug treatment. Together, all the studies referred to above highlight that optical trapping is a sensitive research tool to investigate the cell-cell interactions at the single-cell level and allows tracking the changes in interaction forces caused by a pathological process or a therapeutic intervention.

The methods described above for establishing a single-cell adhesion based on determination of the interaction force in the range from pN to nN were previously applied for various types of human neoplastic cells^20,50,51^. Nonetheless, there is still a lack of effective methods with the potential to provide a basis for reliable and minimally invasive cancer detection. The reason may be that to gain statistically reliable information on cell adhesion, large numbers of cells must be measured. The application of atomic force microscopy and micropipette requires several minutes of 1. cell adhesion to cantilever and 2. direct cell-cell contact time to generate detectable levels of force. The costly and time-consuming atomic force microscopy only allows the processing of a small number of cells in a single experiment; thus, it is not yet sufficient for large-scale experiments. In addition, the manipulation process should ideally also not damage the cells, but still enable maintaining the cell-cell contact, thus, permitting a controlled observation of how the interaction process unfolds. Meanwhile, when the adhesion between two living cells was formed, any of cell could not be picked up without cell membrane rupture during the detachment process, as was evidenced in previous works^21,49,52^.

In our experimental setup, the cell-to-cell adhesion is induced by the mechanical forces and precisely controlled in a time scale. Human mesenchymal stromal cells were used to mimic new contact sites for time-established B-cell adhesion. We have made a significant improvement over population cell adhesion study by elucidating the differences in adhesion between normal and lymphoma cells at early stages. We have reduced the risk of interference in the normal adhesion of B-cells by avoiding any direct mechanical contact with the cells as well as by introducing surface attachments to provide a reaction force which is applied in micropipettes method and AFM. Furthermore, we have proved that laser operation at 1064 nm holds a low risk of optical damage to primary B-cells. We have established that single normal lymphocyte B as well as lymphoma cells can be optically trapped for over 10 minutes using a laser power of 100 mW without showing any signs of cell damage, while the trapping and moving ability were fully maintained. Finally, we have also eliminated the biotin which is known to interfere with cell adhesion^53,54^ and beads from the experimental setup, which are historically used in optical tweezers study on single cell mechanics.

In this work, we have made an effort to apply the minimally invasive techniques during sample preparation to avoid the disruption of primary cells adhesive properties. For this reason, the cells were collected by lymph node/extranodal tumour biopsy fine needle aspiration biopsy (FNAB), as previously described^55^ and immediately frozen. Additionally, magnetic-activated cell sorting (MACS) was used in this study, as the recommended method for cell isolation prior to transplantation- due to a minimal effect on cell viability^56,57^. The models used in this study, the primary B-cells, are circular in shape and grow in a suspension which make them ideal for optical trapping. Importantly, working with cells in suspension eliminates the step of proteolytic detachment of cells in culture from the adherent substrate, what significantly affects the expression of many cell surface antigens resulting in impaired cell’s native adhesion^58,59^.

Finally, our experimental approach was validated using a panel of lymphoma cells obtained from 37 patients with B-NHL and normal B-cells isolated from reactive lymph nodes of 15 non-neoplastic patients. We have determined and compared the *in vitro* adhesive properties of 1087 normal B-cells and 2187 B-NHL cells in optical tweezers, one by one. We observed the overall decrease in lymphoma cell adhesion comparing normal cells. Mean contact time for establishment of native adhesion was 26.7 ± 16.6 s for individual normal B-cell, while for lymphoma cells it was 208.8 ± 102.3 s, p < 0.001. Moreover, by analyzing the adhesive properties of individual lymphoma cells we provided the direct evidences for B-NHL lymphoma heterogeneity. Finally, we demonstrated that the application of optical tweezers in cell adhesion experiments can discriminate with accuracy between the two particular cell types in the seconds to minutes time scale and potentially form a platform for differentiation between normal and neoplastic cell types.

## Experimental

### Experimental setup

**Figure 1:**
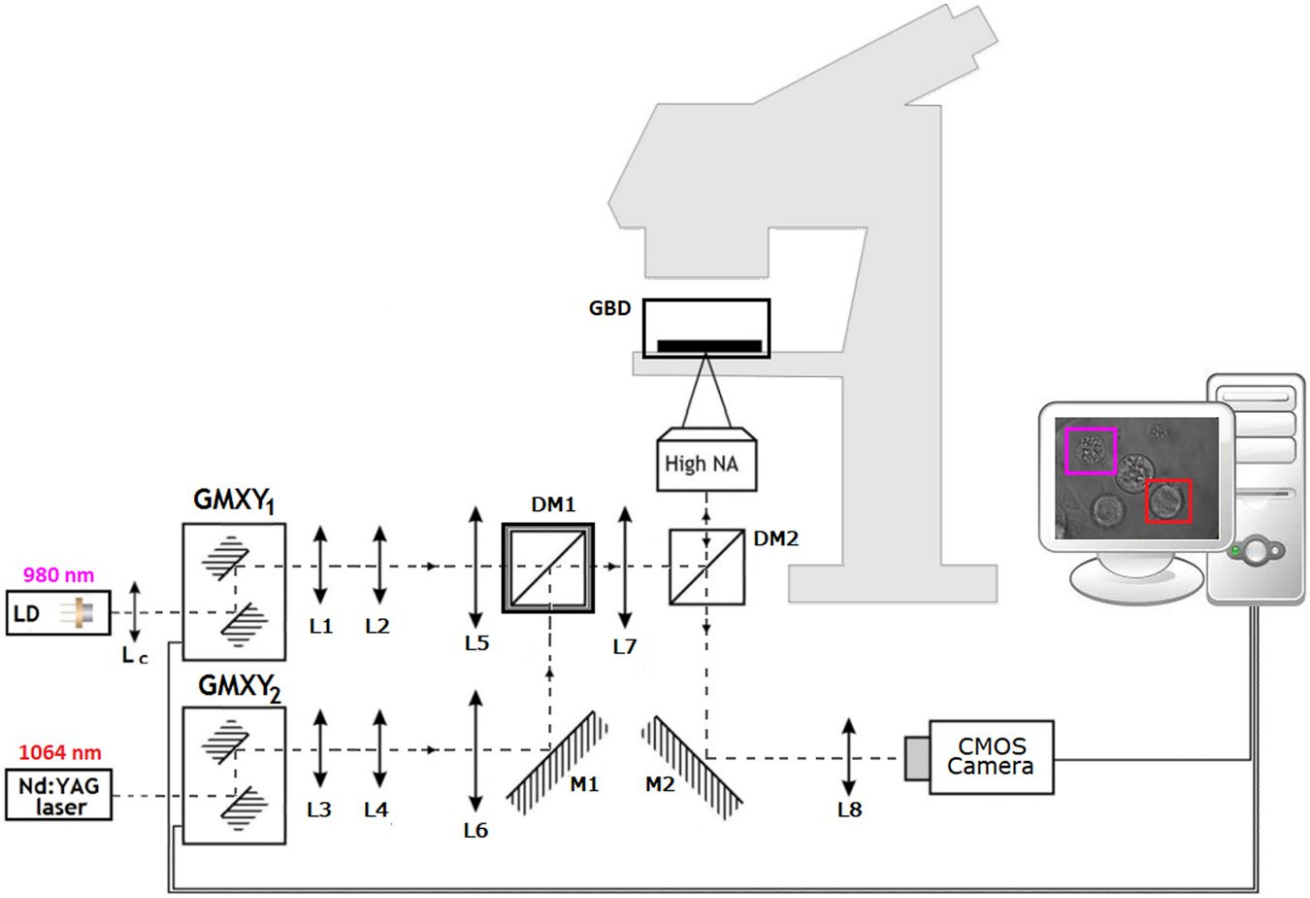
Schematic illustration of the double wavelength optical tweezers setup. Lc = collimating lens; L1 ÷ L8 = lenses; M1, M2 = mirrors; DM1, DM2 = dichroic mirrors; GMXY1, GMXY2 = galvano-mirror XY scanning systems; High-NA = high numerical aperture microscope objective; GBD = glass bottom dish with stromal cells in plexiglass box.

The optical tweezers were previously used by our group in a variety of applications, e.g cell-to-cell adhesion^52,60^, localized hyperthermia^61^, and drug testing^62^. The configuration was adapted to trap and move individual B-cells. Our home-made double wavelength optical tweezers setup is shown in Fig. 1. The system is based on the standard microscope. Optical traps are generated with two laser sources: Nd:YAG 1064 nm laser (max. output power 2.5 W) and laser diode 980 nm with collimating lens Lc. The lenses pair L1-L2 and L3-L4 expand the laser beams. The telescopes L5-L7 and L6-L7 overfill the back aperture of the high NA objective, which creates optical traps in the sample plane of glass bottom dish with stromal cells. The 980 nm laser was used to generate a second appropriately strong optical trap, to help of separation of other cells from the region where B-cells in contact with stromal cells were measured. The DM1 dichroic mirror transmits light below 1000 nm and reflects above this wavelength. The DM2 dichroic mirror transmits light below 805 nm and reflects above this wavelength. Application of two laser with different wavelength and dichroic mirror DM1 enables the effective use the power of each laser. It is more effective than lasers at the same wavelength and 50:50 beamsplitter. The optical traps position in microscopic sample are controlled by GMXY1 and GMXY2 galvano-mirror XY scanning systems. The CMOS camera with lens L8 is used for sample imaging. The entire optical tweezers setup is controlled by home-made software written under C++\CLI for. NET (Microsoft, Warsaw, Poland). Manipulations on single cells were performed within a glass bottom dish. To maintain the temperature above 30 °C throughout the manipulations, the glass bottom dish with stromal cells was placed in a plexiglass box to keep thermal isolation from the surrounding thermal influences and changed every 20 minutes.

### Clinical samples

Lymphoma cells (as the cellular suspension) were obtained by FNAB or by ultrasound-guided-FNAB from the involved lymph nodes/tumors, or from fresh surgery-derived lymphoma tissues harvested for diagnostic purposes. The final diagnosis of diffuse large B-cell lymphoma (DLBCL, NOS), follicular lymphoma (FL), high grade B-cell lymphoma (HGBL), mantle cell lymphoma (MCL), Burkitt lymphoma (BL), primary cutaneous diffuse large B-cell lymphoma (PCDLBCL) and extranodal marginal zone lymphoma (EMZL) was made according to the current 2017 WHO classification^63^, including morphological criteria and immunohistochemical examination, with flow cytometry immunophenotyping, cytogenetics analysis, along with clinical characteristics of patients evaluated by a hematopathologist in all cases, as previously described^55^. The normal B-cells were isolated from normal human lymph nodes of non-neoplastic patients prior to optical tweezers manipulations according to the protocol described in the Material and Methods section. Additionally, detailed flow cytometry analysis of lymphoma samples was made before the study, including the assessment of % of pathological cells (see suppl. Table 1). In total, 2187 primary cells derived from 37 B-NHL patients and 1087 cells obtained from 15 controls were included to study. The Table 1 shows the final diagnosis of clinical samples. Patients age was from 21 to 89 (59.2 ± 15.1 years), while controls from 29 to 89 (61.1 ± 17.8 years). Male to female ratio was 19:18 for patients and 1:2 for controls. Both groups of patients did not differ significantly in their age and gender structures, p = 0.699 and p = 0.449, respectively, as calculated by the Mann-Whitney U test.

**Table 1:** Diagnosis of the clinical samples.

Subtype of lymphoma	No. of patients	Cells [No.]	Controls	No. of controls	Cells [No.]
DLBCL, NOS	13	788	LR	9	693
FL	9	304	CA	4	282
HGBL	5	541	CC	2	112
MCL	5	270	**Total**	**15**	**1087**
BL	2	130			
PCDLBCL	2	112			
EMZL	1	42			
**Total**	**37**	**2187**			

DLBCL, NOS = diffuse large B-cell lymphoma not otherwise specified; FL = follicular lymphoma; HGBL = high grade B-cell lymphoma; MCL = mantle cell lymphoma; BL = Burkitt lymphoma; PCDLBCL = primary cutaneous diffuse large B-cell lymphoma; EMZL = extranodal marginal zone lymphoma; LR = reactive lymphadenopathy; CA = lymphadenopathy associated with cholecystis acute; CC = lymphadenopathy associated with chronic colitis.

### Experimental procedure

**Figure 2:**
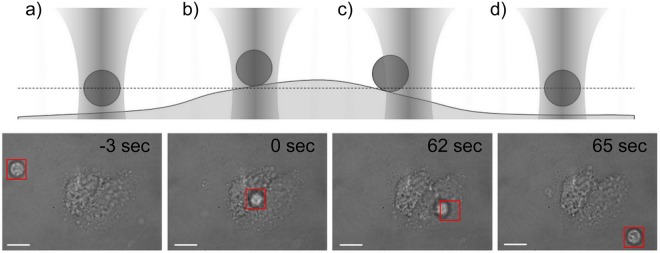
Diagrams (top) and representative microscopic pictures (bottom) of B-cell-mesenchymal stromal cell adhesion measurements. The methodology used to study nascent adhesion is separated into distinct phases. (**a**) The B-cell is optically trapped and located toward mesenchymal stromal cell; (**b**) B-cell is seeded in the central part of stromal cell and held in optical trapp for a determined time; (**c**) an attempt to detach the B-cell from stromal cell is performed to test wheather adhesive interactions are established; (**d**) the B-cell is moved away from stromal cell after cell-to-cell contact was broken at a defined time point. The microscopic images represents testing of single primary non-Hodgkin lymphoma cell adhesion to MSc. The optical trap (red) was used to bring the lymphoma cell into contact with MSC, maintain cell-to-cell interactions and finally testing the establishment of cell adhesion at a defined time point. The scalebar is set at 15 μm.

Cells suspension (2 × 10^4^ cells) was dropped into a glass bottom dish with stromal cells and placed in a plexiglass box onto a motorized stage of the microscope. The time of cell sinking to the glass bottom ranged from 3 to 5 minutes. Optical tweezers were used to target an individual B-cell, optically trap it, and bring it into contact with mesenchymal stromal cell membrane. The optical adhesion of a B-cell to a stromal cell is schematically presented in Fig. 2a–d. In brief, the optical trap (laser power 100 mW) isolated the cell of interest and the microscope stage was moved to deliver it into contact with the central part of a stromal cell. It should be noted that the organization of the cytoskeleton differs on the edge and in the central part of the cell ultimately affecting the adhesive interactions^19^. As soon as cell-cell contact was initiated, the B-cell was retained in optical trap centre to interact with the stromal cell for a given time. The experimentally predefined contact time intervals for cells trapping were 5, 10, 20, 30, 40, 60, 90 s and 40, 60, 90, 120, 150, 180, 210, 240, 270, 300, 360, 420 s for normal B lymphocytes and lymphoma cells, respectively. Next, the optical trap was moved 20 μm away from the interacting cells for 15 s. The formation of the adhesion junction between cells was evidenced by the fact that the B-cell could not be pulled away from the stromal cells by the optical trap during three detachment attempts. If the cellular contact was broken, the B-cell was again held in the optical trap to interact with the stromal cell for a longer time. The B-cell was attached to the stromal cell for a maximum of three times and the entire time of an individual cell manipulation did not exceed 540 s. In our research, a constant laser power was used for primary cells isolated from controls and lymphoma samples despite their significant difference in size. The diffraction-limited spot size for 1064 nm laser and microscope objective 100x is approximately 1um is located in the center of the square marker (Fig. 2a–d) and is invisible because the camera uses an IR filter cutting a strong laser beam. In static situation, when the cell is trapped, it is relocated to the center of focused laser spot (Fig. 2a,b,d). If the cell is moved by the optical tweezers, focused the laser interacts near the cell membrane, not in the centre as in the static case (Fig. 2c). The force coming from the optical tweezers depends on the parameters of the laser beam and also on the properties of the trapped object^37^. The decisive moment in this measurement is pulling the cell away from the stromal cell. In this point, the acting force is constant regardless of the cell’s size, because the focused laser beam interacts only in the vicinity of the cell membrane^38^. Thus, normal cells at about 7 μm size and lymphoma cell (12–14 μm) were attached and then pulled away from the stromal cells with the same force. The precise measurements of the adhesion force we described in our earlier paper^60^.

## Results and Discussion

### The assessment of the influence of the laser beam on living cells viability

**Figure 3:**
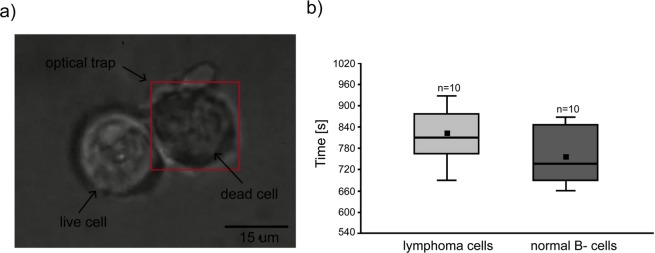
(**a**) Trypan blue accumulation on the surface of untreated DLBCL cell (left), while the dead cell was held in optical trap > 800 s at 100 mW of laser power what resulted in cell membrane rupture and entering the dye inside the cell. The red frame indicates the area of operating range of the optical trap, while the focused laser beam is located in the centre of the trapped cell. The scalebar is set at 15 μm. (**b**) Comparison of the normal and lymphoma primary cells viability exposed to laser power of 100 mW using Trypan blue. Manipulation over 756 ± 73.24 s and 819 ± 72.31s for normal and lymphoma cells, respectively damaged the cell membrane.

The optically induced damage to the cell depends mainly on the wavelength of the trapping beam, the exposure time, and the cell type that is investigated^64^. Here, we applied the previously described method^52,61^ using Trypan blue to validate the influence of laser power on the cell membrane rupture resulting in cell destruction. We noted the distinct accumulation of the dye on the surface of the untreated cells, while optical trapping of cells under experimentally established laser power and time resulted in cell membrane rupture, entering the dye inside the cell and caused the cytoplasm to darken (Fig. 3a). Based on the results achieved with commercial DLBCL cell lines where laser power of 100 mW, 200 mW, 300 mW and 400 mW at the entrance of the inverted microscope was applied, we established the laser power of 100 mW with minimal influence on cell morphology and viability.

To investigate the influence of the laser beam of 100 mW on the primary cell viability, we optically manipulated a total of 10 normal B-cells and 10 lymphoma cells originating from two control donors and two lymphoma patients, respectively. Here it was shown that the use of laser power of 100 mW allows for non-invasive laser exposure over 540 s, which was the maximum manipulation time on an individual cell in this study. We established that the mean time of normal B-cells disruption under laser power of 100 mW was 756 ± 73.24 s and ranged from 660 to 870 s. Lymphoma cell membrane rupture was observed after 819 ± 72.31 s and it ranged from 690 to 930 s (Fig. 3b). Additionally, during the interval time of exposure < 600 s, no changes in the cell morphology provoked by the optical tweezers were observed. We have not found any significant differences between the normal and lymphoma cell viability under the above conditions (p = 0.083, Student’s -t-test). In turn, the comparison of the data obtained in the present study for primary cells with those obtained previously for lymphoma cell lines^52^ indicates that primary cells are more resistant to 100 mW laser exposure under our experimental conditions.

### Adhesion of normal and lymphoma B-cells to MSC at a single- cell level

**Figure 4:**
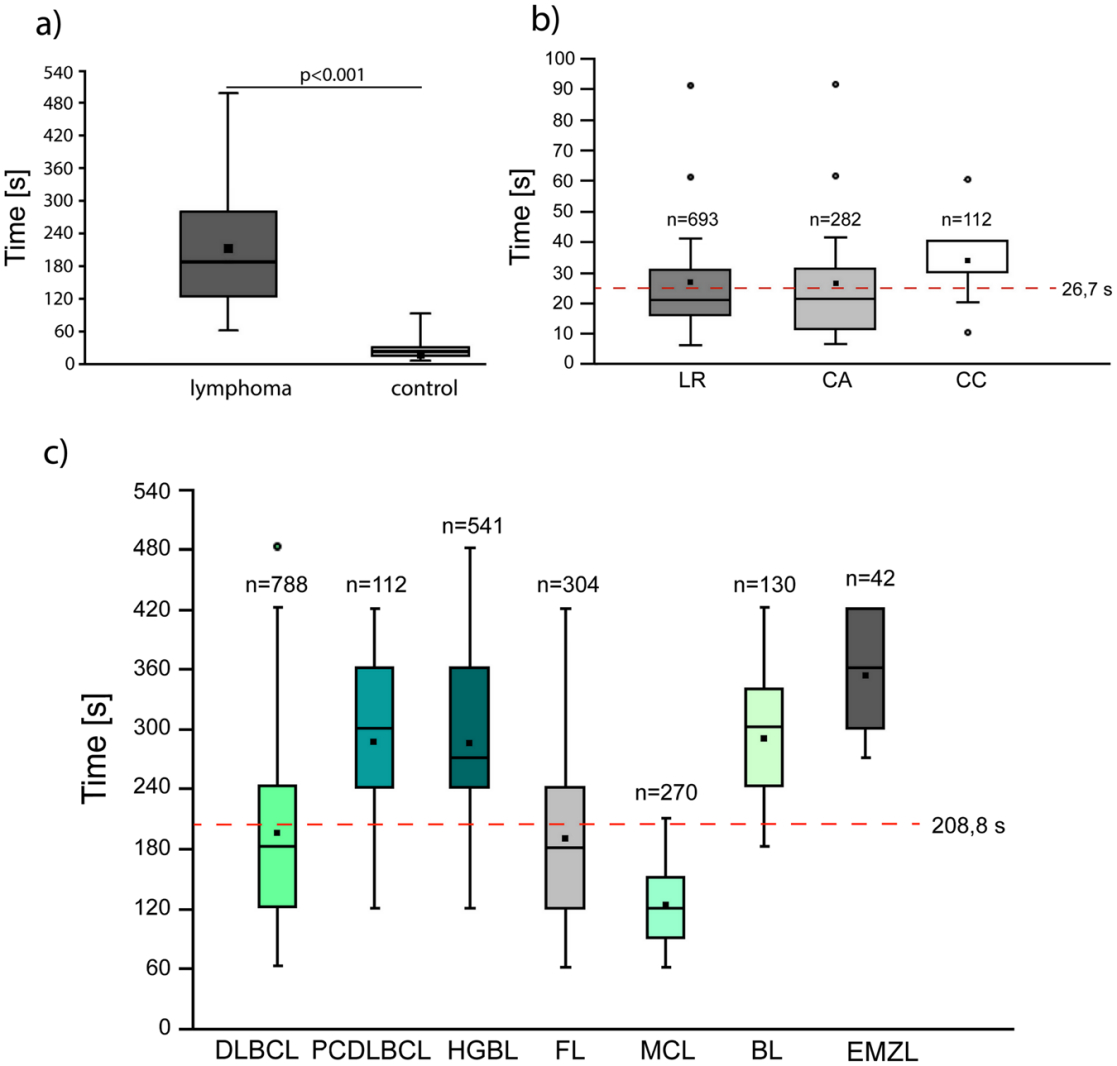
(**a**) Box and whiskers plot of differences in normal and lymphoma single- cell adhesion to mesenchymal stromal cells, p < 0.001, Mann-Whitney U test. (**b**) Single- cell adhesion for normal B-cells and most common B-cell non-Hodgkin lymphoma subgroups (**c**). The symbol (°) indicates outliers, while the red dotted line means the average time required to form a stable connection to MSCs.

Here, on the basis of the results obtained for commercial lymphoma cell lines^52^, we have investigated adhesive properties of single B-NHL cells and normal B-cells to mesenchymal stromal cells in the regime of the direct contact time. The clinical samples were obtained from 37 lymphoma patients and 15 control donors (Tables 2 and 3). The number of cells examined for each individual patient varied from 42 to 80 for lymphoma patients and from 48 to 110 for controls. Those differences resulted from the variable quantity of the clinical material obtained for the study and the time required to perform a single experiment. The total number of cells probed by the optical tweezers was n = 2187 for lymphoma cells and n = 1087 for normal B-cells. We have determined on this large cohort of samples that lymphoma cells exhibit significantly reduced adhesive properties compared to non-neoplastic B-cells, Fig. 4a. The mean time required for establishment of adhesive interactions between an individual normal B-cell and the mesenchymal stromal cell was 26.7 ± 16.6 s, while for lymphoma cells it was 208.8 ± 102.3 s, p < 0.001. This observation is in line with the first work where the optical tweezers were applied to the investigation of cell-cell adhesion in time scale^21^. The authors provided direct evidence that the neural stem cell adhesion dynamics proceeds in time scale of only a few second (~5 s). Next, the same research group has used the above approach to study the adhesive properties of the human neuroblastoma SK-N-SH and rat C6 glioma cells^22^. The authors have proved that the average minimum time for adhesion to occur for tumour cells substantially increases to ~20–25 s, in some cases up to 45 s, indicating significant decrease in cell-cell adhesion. Our results are also in good agreement with the study on the single primary T-cell adhesion in optical tweezers, where the time required to obtain cell-cell initial interaction was between 30–120 s^49^. Another recently published work of Xie *et al*. reports the adhesion dynamics of a single breast carcinoma cell to the bone marrow endothelial cell using atomic force microscope which is from 0.5 to 300 s^50^. Importantly, both in our research and in the above-mentioned works, the range of single cell-cell adhesion time was rather wide, reflecting cellular heterogeneity. In our experimental work, the contact time required for cell-cell adhesion to occurred ranged from 5 to 90 s and from 60 to 480 s for normal B-cells and lymphoma cells, respectively. Thus, we observed that to describe the primary B-cells, highly adhesive phenotype and low adhesive phenotype should be considered.

**Table 2:** The average contact time required for the formation of adhesion between B-cell non-Hodgkin lymphoma cells and mesenchymal stromal cells in optical tweezers, N = number of manipulated cells; SD = standard deviation.

No.	Diagnosis	N	Mean adhesion ± SD [s]	Time range [s]	No.	Diagnosis	N	Mean adhesion ± SD [s]	Time range [s]
1.	DLBCL, NOS	73	131.5 ± 49.2	60–210	20.	HGBL	67	265.1 ± 94.8	120–420
2.	DLBCL, NOS	64	162.2 ± 59.2	90–270	21.	FL	62	202.2 ± 64	120–360
3.	DLBCL, NOS	53	147.2 ± 42.9	90–240	22.	FL	54	120.5 ± 45.3	60–240
4.	DLBCL, NOS	80	157.9 ± 57.3	90–300	23.	FL	56	304.8 ± 59.4	210–420
5.	DLBCL, NOS	76	168.9 ± 55.8	90–270	24.	FL	65	180 ± 50.3	90–270
6.	DLBCL, NOS	54	160.5 ± 48.4	90–300	25.	FL	72	109.2 ± 32.4	60–180
7.	DLBCL, NOS	47	327.8 ± 59.5	210–420	26.	FL	55	317.4 ± 86.1	120–420
8.	DLBCL, NOS	60	339.5 ± 65.4	210–480	27.	FL	53	110.9 ± 28.9	60–150
9.	DLBCL, NOS	59	163.2 ± 44.7	90–300	28.	FL	57	201.6 ± 69.4	120–360
10.	DLBCL, NOS	53	162.4 ± 43	120–360	29.	FL	68	157.1 ± 60.9	60–300
11.	DLBCL, NOS	52	264.2 ± 52.1	120–360	30.	MCL	61	119 ± 46	60–180
12.	DLBCL, NOS	65	167.1 ± 49.7	90–300	31.	MCL	51	120 ± 32.3	60–180
13.	DLBCL, NOS	52	209.4 ± 54.3	120–300	32.	MCL	50	115.2 ± 41.7	60–210
14.	PCDLBCL	64	257.8 ± 68.2	120–360	33.	MCL	50	135.3 ± 30.5	90–180
15.	PCDLBCL	47	324.2 ± 59.2	210–420	34.	MCL	58	124.1 ± 33.6	60–180
16.	HGBL	58	300.5 ± 61.4	180–420	35.	BL	72	268.7 ± 62.9	180–420
17.	HGBL	54	336.7 ± 85.2	180–480	36.	BL	58	315 ± 73.9	180–420
18.	HGBL	58	282.9 ± 62.6	180–420	37.	EMZL	42	350.7 ± 53.8	270–420
19.	HGBL	67	256.6 ± 87.1	120–420

**Table 3:** The average contact time required for the formation of adhesion between normal B-cells and mesenchymal stromal cells in optical tweezers, N = number of manipulated cells; SD = standard deviation.

No.	Diagnosis	N	Mean adhesion ± SD [s]	Time range [s]
1.	CA	93	17.1 ± 9.7	5–30
2.	CA	74	18.5 ± 8.1	5–40
3.	CA	67	29.6 ± 12.1	10–60
4.	CA	48	41.2 ± 22.5	10–90
5.	CC	55	33.4 ± 13.9	10–60
6.	CC	57	49.8 ± 17	20–90
7.	LR	98	18.4 ± 9.9	5–60
8.	LR	110	22.4 ± 13.1	5–60
9.	LR	98	16.7 ± 8.3	5–40
10.	LR	54	37.2 ± 21.3	10–90
11.	LR	78	36.2 ± 20	10–90
12.	LR	68	23.8 ± 11.7	5–60
13.	LR	51	42.3 ± 11.1	30–60
14.	LR	70	21.8 ± 7.2	10–40
15.	LR	66	20.4 ± 10.3	5–40

### The adhesive properties of the most common B-NHL subtypes at a single-cell level

B-cell non-Hodgkin lymphomas are clinically and biologically very heterogeneous, their tumour cells are the malignant counterparts of normal B-lymphocytes arrested at specific stages of maturation^65^. Therefore, we asked whether B-NHL subtypes differ in adhesive properties to the marrow stromal cell at a single-cell level.

First, we have observed that normal B-cells isolated from lymph node of patients with different final diagnosis did not differ significantly in adhesive properties, Ryc. 4b. In turn, the Fig. 4c shows that the average time of nascent adhesion formation of lymphoma primary cells to stromal cells was from 122.7 ± 37.8 to 350.7 ± 53.8 for mantle cell lymphoma (MCL) and extranodal marginal zone lymphoma (EMZL), respectively. We have also noted that the adhesive properties of both MCL and EMZL cells differed significantly from the other lymphoma subtypes involved in the study, p < 0.01. The high adhesive properties of mantle cell lymphoma cells to MSCs are supported by the previous retrospective studies showing that the MCL more frequently involves the peripheral blood and bone marrow compared to the other B-NHLs^66,67^. Finally, in this work, the cell-cell adhesion was established after different contact times with a 60 s minimum for DLBCL, FL, and MCL and 480 s maximum time frame for DLBCL and HGBL cells. Thus, DLBCL cells exhibited the greatest variability in adhesive properties of individual cells among all subgroups of lymphoma included in the study. At the same time, the adhesive properties of DLBCL cells differed significantly from the other B-NHL subtypes (p < 0.01), except for FL (p = 0.056). Observed differences in the adhesive properties of B-NHLs are most likely related to differential expression of various adhesion molecules on the membrane of lymphoma cells^68–70^ as well as altered cell-cell and cell-extracellular matrix communication^71,72^. In the case of lymphoproliferative tumours, there are many unknowns regarding cell adhesive molecules, particularly the role of mechanical and chemical signals generated by them to facilitate the crosstalk between cell and extracellular environment. These aspects, although interesting, are beyond the scope of the present study and need to be proved in an independent study on the blocking of a specific adhesion protein by antibodies and evaluating nascent adhesion formation in optical tweezers as well as performing standard bulk adhesion assays. This observation may be valuable for the further research that may be useful to enhance our understanding of lymphoma cells heterogenicity. Thus, as a next step, the molecular background of the wide distribution of time-dependent adhesion in the same clinical sample will be explored using the optical tweezers combined with fluorescent evaluation of adhesion proteins expression.

### Analysis of the single-cell adhesion in regard to clinicopathological features

**Figure 5:**
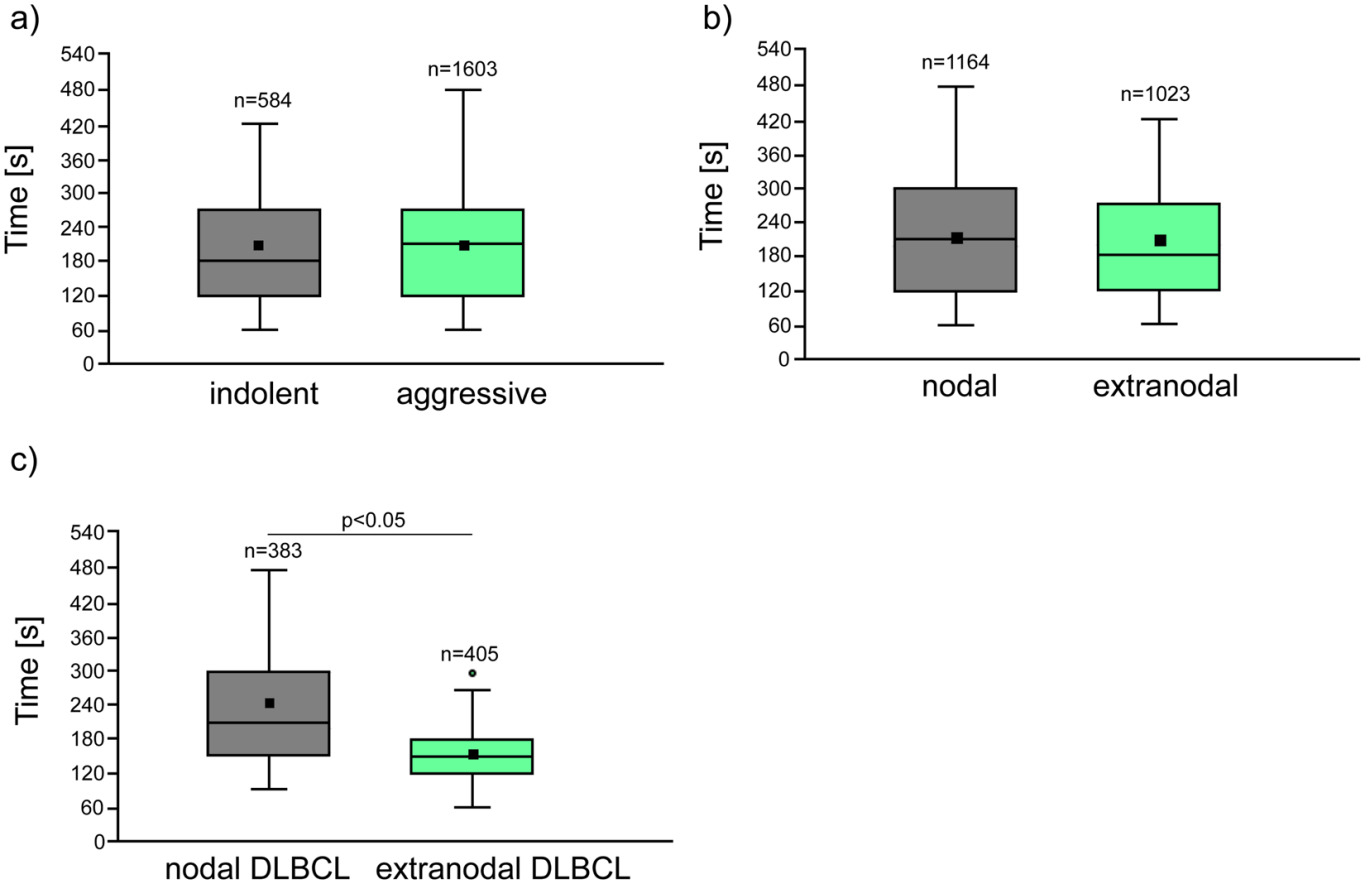
Box and whiskers plot of time-dependent single- cell adhesion to marrow stromal cells. (**a**) Primary indolent and aggressive lymphoma subtypes. Indolent lymphomas included FL and EMZL when aggressive lymphomas included all lymphomas except for indolent. (**b**) All manipulated cells classified according to the nodal vs. extranodal presentation. (**c**) DLBCL cells classified according to the nodal vs. extranodal presentation, the Mann-Whitney U test. The symbol (°) indicates outliers. The square markers denote the mean.

Our data revealing heterogenicity in adhesion to MSC in non-Hodgkin lymphoma subgroups also raised the questions whether differential adhesive properties may be associated with the disease stage. Primary cells examined in our study derived from indolent lymphomas (FL and EMZL) formed adhesion contacts with marrow stromal cells in 198.5 ± 99.0 s, while cells derived from aggressive lymphomas formed them in 212.6 ± 95.90 s (Fig. 5a). Our findings prove that there is no direct relationship between lymphomatous cells adhesive capabilities and their aggressiveness. In our model, mantle cell lymphoma cells showed a highly adhesive phenotype, while this neoplasm is known for its aggressiveness and tendency to disseminate. In contrast, other aggressive tumours like HGBL and BL had significantly higher adhesion times. Moreover, typically indolent EMZL showed the highest adhesion time in our experiment.

Non-Hodgkin lymphomas may arise in nearly any extranodal sites (termed as primary extranodal lymphomas) and the number of patients with extranodal NHLs is rapidly increasing^73,74^. Hematogenous spread of the disease from lymph nodes (primary nodal lymphoma) to extranodal tissue is referred to as a secondary extranodal lymphoma. Extranodal disease is prognostically important in any NHL lymphoma. The large-scale database studies on NHL shows for the first time that primary extranodal disease tends to present at an earlier stage than the primary nodal disease^5^. More contemporary reports say that not only the nodal or extranodal presentation, but also the involvement of specific sites, may be associated with particular clinicobiological characteristics and disease prognosis^74,75^. Thus, based on our experimental data, we have asked whether individual primary cells obtained from nodal and extranodal lymphoma samples differ in adhesive properties. We have established that the contact time required for cell-cell adhesion to occur was similar for all B-NHL (p = 0.749) and equals to 207.7 ± 77.9 s and 215.4 ± 84.4 s for cells derived from extranodal and nodal samples, respectively, Fig. 5b. Nevertheless, as presented in Fig. 5c, the adhesive properties within DLBCL subgroup were considerably different (p = 0.038) and estimated as equal to 155.1 ± 53.9 s and 231.2 ± 90.0 s for cells derived from extranodal and nodal samples, respectively. These findings may be related to diversities in local microenvironment of nodal and extranodal DLBCL or to some clinical features of the disease like the capacity for local infiltration and dissemination to the other sites.

Results obtained in our study may be particularly important considering that the recently extensively studied genetic and molecular alterations indicate that some of extranodal DLBCLs may be distinct disease entities with their own prognosis and risk of recurrence^75,76^. Because of the limited number of samples belonging to distinct NHL subgroups, this comparison was made for DLBCL only.

### Diagnostic potential of our approach to the B- cells differentiation from clinical samples

**Figure 6:**
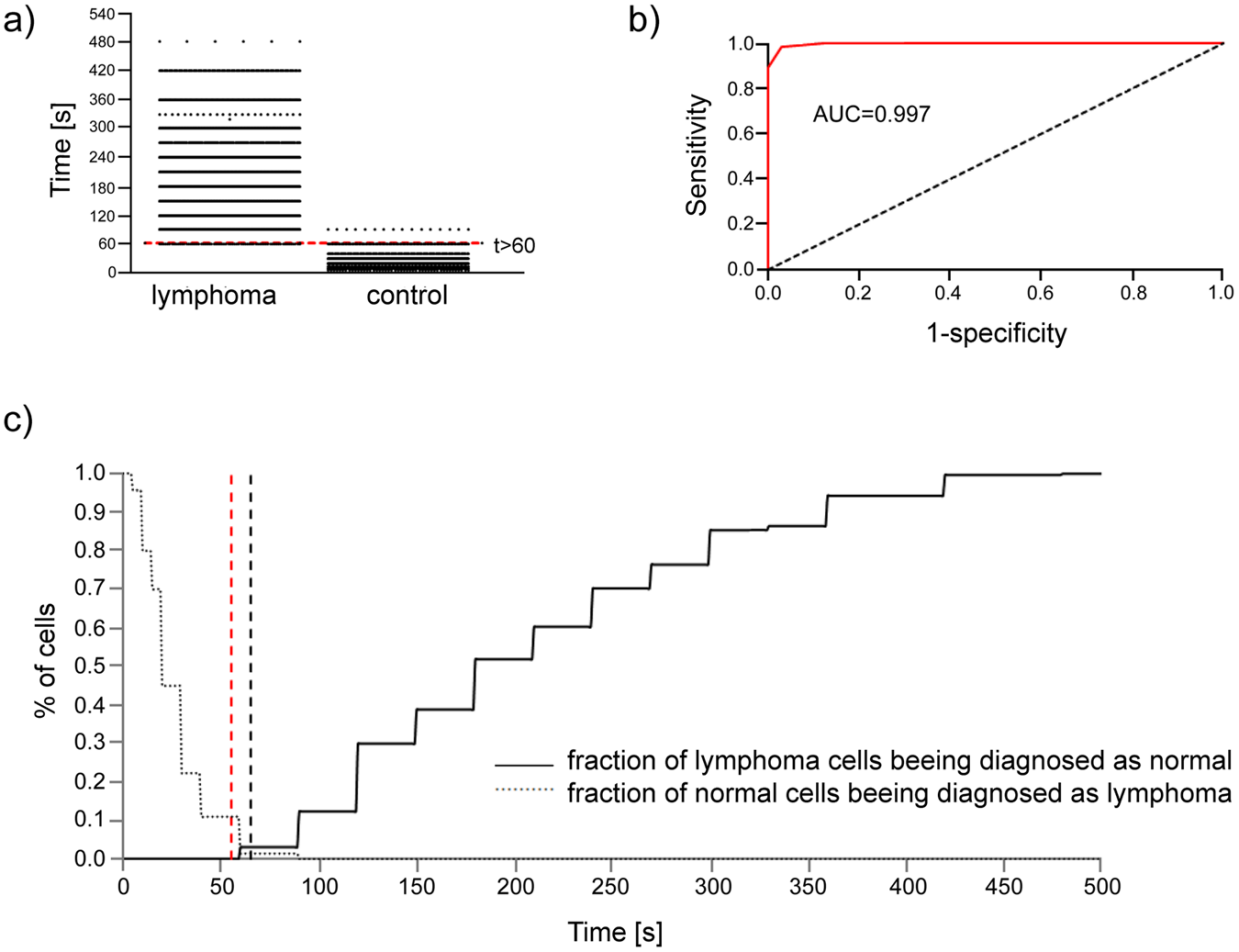
(**a**) Frequency distribution of cell-adhesion time for lymphoma cells and the normal ones. The red line denotes the cut-off value, which minimizes the fraction of false classification. (**b**) ROC curve as discriminator between lymphoma and normal B-cells. AUC = area under the ROC curve. The optimal cutoff value is t > 60 s, for which the probability of false negative result is 0.0311 while for the false positive 0.0147. (**c**) Fractions of misclasifications of lymphoma cells (solid line) and normal cells (dotted line). The black dashed line denoted the cut-off line that minimizes the total number of misclassification. The red dahed line denoted the cut-off value that would eliminate the misclassification of lymphoma cells within the sample.

Next, we have asked if the B-cell-to-mesenchymal stromal cell adhesion can be the mean to distinguish lymphoma cells from the normal ones. To address this question, we analysed the contact time of cell adhesion for individual malignant and normal cells separately (Fig. 6a). The aim was to find the cut-off value that would best distinguish the lymphoma from the normal cells. Amongst 2187 lymphoma cells, for 68 cells, the contact time was equal to 60 s. Amongst 1087 normal cells, for 16 cells, the contact time was greater than 60 s. Basing on the ROC curve, the optimal cut-off value that minimises the fraction of misclassification is t > 60 s (Fig. 6b). For this cut-off value, there is 0.0311 chance of diagnosing malignant cell as normal and 0.0147 of diagnosing a normal cell as a malignant one. In clinical practice, false negative and false positive might be, however, not equally treated. The Fig. 6c presents these fractions as functions of a cut-off value. It can be seen that within the examined sample, the fraction of cells wrongly diagnosed as normal would be zero for cut-off value t >= 60, s while the fraction of cells wrongly diagnosed as pathological would be zero for the cut-off t > 90 s. In practice, more serious implications are caused by wrongly diagnosing a sick person as a healthy one; therefore, it would be safer to adopt the value t >= 60 s as a criterion for classifying the disease, with the contact time of 60 s as requiring further examinations.

The ambiguity appears only for cell-to-cell contact times between 60 and 90 s. It would be recommended to examine more cells if the result for a single cell falls within this range. However, a general rule would be preferred: how many cells should be examined to get a given percent of certainty. To estimate such a number, it would not be proper just to treat the whole sample of 2187 lymphoma cells as a whole, because the fraction of cells with time equal to 60 s (thus leading to misclassification) is distributed highly inconsistently amongst patients. Among 37 patients only for 9 there were observed cells with time equal to 60 s, and the fraction of such “ambiguous” cells for particular patients ranged from 0 to 20%. Of course, as the sample of neoplastic tissue is not large, there is no guarantee that for another lymphoma patient, there will not be one with this fraction at, e.g., 0.5. We may try to fit the distribution of this fractions with some theoretical distribution. It turns out that the data may be approximated with exponential distribution with the parameter 23.9075 and testing the hypothesis that the data is indeed distributed according to this distribution is verified with p = 0.001. Thus, we can calculate the probability that within a sample of cells taken from a given patient there will be a given fraction of “ambiguous” cells. For any fitted probability density function, the probability that for k-cells sample (for a given person) all of them will be “ambiguous” (if at least one is not, the examination could be regarded as conclusive) can be calculated as the k-th central moment of this approximating probability density function. For the above-mentioned exponential distribution with shape parameter 23.9075, those probabilities are as listed in Table 4.

**Table 4:** Probability of false classification of a lymphoma sample as a normal one for the given number of cells tested in optical tweezers.

No. of cells	Probability of false classification	No. of cells	Probability of false classification
1.	0.041828	11.	2.73E-08
2.	0.003499	12.	1.37E-08
3.	0.000439	13.	7.39E-09
4.	7.35E-05	14.	4.28E-09
5.	1.54E-05	15.	2.65E-09
6.	3.86E-06	16.	1.73E-09
7.	1.13E-06	17.	1.19E-09
8.	3.78E-07	18.	8.53E-10
9.	1.42E-07	19.	6.37E-10
10.	5.94E-08	20.	4.91E-10

## Conclusions

Here, we have reported the first evidence of a difference in the adhesive properties of individual normal B-cells and non-Hodgkin lymphoma cells using optical tweezers. Our proof of concept experimental results showed that the normal and lymphoma cells display different adhesive properties. This finding encouraged us to adapt the optical trapping setup to detection of lymphoma cells by evaluating a single- cell adhesion to a mesenchymal stromal cell in a time scale on a large number of clinical samples. Eventually, the method has been tested using primary cells taken from 15 healthy individuals and 37 patients with B-NHL. The method has shown the ability to identify lymphoma cells unambiguously and we believe that predicting the adhesion abnormalities in a clinical sample might potentially help in fast and low-invasive pre-stratification of patients prior to a treatment. Moreover, we have shown that studying interactions between lymphoma and stromal cells in optical tweezers has a potential in aiding our understanding of heterogenicity of B-NHL by systematically initiating and tracking the process of adhesion formation from the beginning to the end, one cell pair at a time, with precision unattainable to traditional approaches to analysing cell-cell adhesion.

## Material and Methods

### Clinical samples

Samples were collected under Wrocław Medical University ethics comittee approval (KB-504/2014, 1 Oct. 2104) after informed consent process in accordance with the Declaration of Helsinki. Lymph node aspirates or surgically resected lymph nodes were taken from patients and controls with suspected lymphoproliferation or undergoing gastrointestinal surgery. Detailed flow cytometry analysis was made before the study for each clinical sample, including assessment of the % of pathological cells. Clinical characteristics of the lymphoma patients and donors enrolled to the study, origin of the samples, sampling method as well as flow cytometric assessment of sample composition are listed in supplementary Tables 1 and 2.

Surgically removed lymph nodes were washed with phosphate buffered saline (PBS, Thermo Fisher Scientific, Berlin, Germany), cut and mechanically dissected by nylon mesh with 70 μm pores (Corning, Wiesbaden, Germany). Fine needle aspiration biopsy was collected to a collection tube filled with 10 mL of cold PBS. Both surgery and biopsy derived specimens with PBS were transferred to K_2_EDTA tubes (EDTA 7.2 mg, Becton Dickinson, Heidelberg, Germany) for 20 minutes and frozen in 30% foetal bovine serum (FBS, Thermo Fisher Scientific) and 10% dimethyl sulfoxide (DMSO, Sigma-Aldrich) for further studies. Samples were transported and stored in liquid nitrogen.

### Primary cells isolation via magnetic-activated cell sorting (MACS)

Prior to the measurements in optical tweezers, biopsy specimens were thawed, centrifuged at 2000 RCF x 10 min, resuspended in RPMI-1640 medium (Thermo Fisher Scientific) with 1% Primocin (InvivoGen, Toulouse, France) and kept in suspension overnight at 37 °C in 5% CO_2_. After sample purification with Ficoll- Paque (Sigma-Adrich, Steinheim am Albuch, Germany), the cells were washed with PBS, incubated with anti-CD3 microbeads (Miltenyi Biotech, Bergisch Gladbach, Germany) and loaded onto a MACS column (Miltenyi Biotech) according to the supplier instructions. The T-cells labelled with anti-CD3 beads were retained in the magnetic field, while the unlabelled B-cells flowed through the column. Negative fraction containing B-cells was collected, washed twice with PBS, resuspended in fresh RPMI and incubated 2 h at 37 °C before OT manipulations. Since the isolated cells were exposed to physical stress during magnetic separation, the viability of primary B-cells was evaluated by Trypan blue dye exclusion assay on the Countess Automated Cell Counter (Thermo Fisher Scientific). Cell preparations after isolation were routinely >95% viable.

Additional isolation with anti-CD5 and anti-CD10 microbeads (Miltenyi Biotech) was used for patients no. 6, 9, 11, 13, 23, 26, 28 and 29 due to the high percentage of normal B-cells in the sample, according to the supplementary Table 1. Ultimately, the sample purity was consistently over 95% of CD19 positive B-cells exhibiting malignant phenotype what was confirmed for selected samples by flow cytometry.

### Mesenchymal stromal cells preparation

Human mesenchymal stromal cell line HS-5 (American Type Culture Collection (ATCC), Manassas, VA, USA) was cultured in Dulbecco’s modified Eagle’s medium (DMEM, Thermo Fisher Scientific) supplemented with heat-inactivated 10% FBS and 1% L-glutamine (Thermo Fisher Scientific) under standard conditions. When the confluence of cells reached 80%, they were detached with 0.25% trypsin (Sigma-Aldrich), counted and 2 × 10^4^ of cells were seeded on a glass bottom dish (Greiner bio-one, Frickenhausen, Germany). On the next day, the medium was replaced. The stromal cells were grown approximately 72 h prior to manipulations in optical tweezers. One hour before the manipulations in optical tweezers, cells not adhered to the glass were washed off with warm DMEM. HS-5 cells were used for experimentation at passage 4–10.

### Statistical analysis

All data are expressed as a mean ± standard deviation (SD). Significance of differences between cell-adhesion time for different groups of cells was determined using the Mann Whitney U test. P-values were given in the text and p < 0.05 was considered as statistically significant. All analysis was performed using Statistica software (Statsoft, Warsaw, Poland).

## Supplementary information

Dataset 1
