# Supplementary material for: Differentiation of single lymphoma primary cells and normal B-cells based on their adhesion to mesenchymal stromal cells in optical tweezers
Source: Sci Rep. 2019 Jul 8;9:9885. doi: 10.1038/s41598-019-46086-y (PMC6614388; doi:10.1038/s41598-019-46086-y)
Supplement: Supplementary file 1 — Dataset 1 [file 41598_2019_46086_MOESM1_ESM.pdf]

Differentiation of single lymphoma primary cells and normal B-cells based on their adhesion to mesenchymal stromal cells in optical tweezers.

Kamila Duś-Szachniewicz[1*], Sławomir Drobczyński[2], Marta Woźniak[1], Krzysztof Zduniak[1], Katarzyna Ostasiewicz[3], Piotr Ziółkowski[1], Aleksandra K. Korzeniewska[2], Anil K. Agrawal[4], Paweł Kołodziej[5], Kinga Walaszek[1], Zbigniew Bystydzieński[6] and Grzegorz Rymkiewicz[6].

[1]Department of Pathology, Wrocław Medical University, Marcinkowskiego 1, 50-368 Wrocław, Poland

[2]Department of Optics and Photonics, Wrocław University of Science and Technology, Faculty of Fundamental Problems of Technology, Wybrzeże Wyspiańskiego 27, 50-370 Wrocław, Poland

[3]Department of Statistics, Wrocław University of Economics, Komandorska 118/120, 53-345 Wrocław, Poland

[4]Division of Pathology, Sokołowski Hospital Wałbrzych, Sokołowskiego 4, 58-309 Wałbrzych, Poland

[5]2nd Department of General and Oncological Surgery, Wrocław Medical University, Borowska 213, 50-556 Wrocław, Poland

[6]Flow Cytometry Laboratory, Department of Pathology and Laboratory Diagnostics, Maria Sklodowska-Curie Institute-Oncology Centre, Wilhelma Konrada Roentgena 5, 02-781 Warsaw, Poland.

*Corresponding author's e-mail: kamila.dus-szachniewicz@umed.wroc.pl

No.	Sex/Age	Diagnosis	Presentation Extranodal vs Nodal	Material	Site of involvement/ Source of FNAB / surgical specimen	Composition of cell suspension by FCM
**1.**	M/50	**DLBCL, NOS, non-GCB**	E	FNAB	cLN	42% of B-NHLs, 55% of T, 2% of M, 1% of NK
**2.**	M/75	**DLBCL, NOS, non-GCB**	E	FNAB	stT	56% of B-NHLs, 3% of B, 26% of T, 3% of NK, 12% of D
**3.**	M/39	**DLBCL, NOS non-GCB**	E	FNAB	ingLN	52% of B-NHLs, 38% of T, 2% of B, 8% of M
**4.**	F/46	**DLBCL, NOS, GCB**	E	FNAB	cLN	96% of B-NHLs, 3% of T , 1% of NK
**5.**	F/61	**DLBCL, NOS, GCB**	E	FNAB	ingT	91% of B-NHLs, 1% of B, 7% of T, 1% of NK
**6.**	F/54	**DLBCL, NOS, GCB**	N	FNAB	cLN	41% of B-NHLs, 5% of B, 48% of T, 5% of M, 1% of NK
**7.**	F/88	**DLBCL, NOS, GCB**	N	FNAB	cLN	87% of B-NHLs, 9% of T, 3% of NK, 1% of M

**8.**	M/62	**DLBCL, NOS, GBC**	N	FNAB	cLN	63% of B-NHLs, 1% of B, 28% of T, 2% of NK, 6% of M
**9.**	F/55	**DLBCL, NOS, GCB**	E	FNAB	abT	24% of B-NHLs, 9% of B, 57% of T, 10% of M
**10.**	M/44	**DLBCL, NOS, GCB**	N	FNAB	cLN	34% of B-NHLs, 3% of B, 60% of T, 3% of NK
**11.**	F/54	**DLBCL, NOS, non- GCB**	N	FNAB	abLN	50% of B-NHLs, 8% of B, 38% of T, 4% of NK
**12.**	M/32	**DLBCL, NOS, intermediate between GCB and non- GCB**	N	FNAB	cLN	23% of B-NHLs, 3% of B, 65% of T, 5% of NK, 4% of M
**13.**	F/61	**DLBCL, NOS, non- GCB**	N	FNAB	cLN	24% of B-NHLs, 7% B, 65% of T, 3% of M, 1% of NK
**14.**	F/74	**PCDLBCL leg type,**	E	FNAB	skinT of the leg	45% of B-NHLs, 3% of B, 23% of T, 15% of M, 14% of NK
**15.**	F/73	**PCDLBC, leg type**	E	FNAB	skinT of the leg	13% of B-NHLs, 58% of T, 19% of NK, 10% of M
**16.**	F/84	**HGBL, NOS, between GCB and non- GCB**	E	FNAB	nasal cavityT	44% of B-NHLs, 2% B, 41% of T, 9% of NK, 4% of M
**17.**	M/37	**HGBL, NOS, GCB**	N	FNAB	cLN	66% of B-NHLs, 1% of B, 28% of T, 5% of NK
**18.**	M/59	**HGBL, NOS, GCB**	E	FNAB	st of left forearm	75% of B-NHLs, 5% of B, 12% of T, 5% of M, 3% of NK
**19.**	M/71	**HGBL, NOS, non- GCB**	E	FNAB	stT	70% of B-NHLs, 23% of T, 4% of NK, 3% of M
**20.**	M/60	**HGBL, NOS**	E	FNAB	skinT	97% of B-NHLs, 2.5% of T, 0.5% of NK
**21.**	F/70	**FL**	E	FNAB	abT	86% of B-NHLs, 5% of B, 8% of T, 1% of NK
**22.**	F/79	**FL**	N	FNAB	abLN	65% of B-NHLs, 4% of B, 30% of T, 1% of NK
**23.**	F/49	**FL**	N	FNAB	cLN	24% of B-NHLs, 3% of B, 67% of T, 3% of M, 3% of NK
**24.**	F/66	**FL**	N	S	abLN	86% of B-NHLs, 14% of T
**25.**	M/65	**FL**	N	S	cLN	53% of B-NHLs, 46% of T, 1% of NK
**26.**	M/56	**FL**	N	FNAB	ingLN	42% of B-NHLs, 7% of B, 46% of T, 3% of NK, 2% of M
**27.**	M/32	**FL**	E	FNAB	abT	95% of B-NHLs, 5% of T
**28.**	M/70	**FL**	N	FNAB	cLN	53% of B-NHLs, 19% of B, 26% of T, 2% of NK
**29.**	F/67	**FL**	N	S	cLN	47% of B-NHLs, 14% of B, 37% of T, 1% of NK, 1% of M
**30.**	F/86	**MCL**	N	FNAB	abLN	96% of B-NHLs, 4% of T
**31.**	F/69	**MCL**	N	FNAB	cLN	86% of B-NHLs, 0.5% of B, 10% of T, 2,5% of NK, 1% of M

**32.**	M/UK	**MCL**	E	S	gastric and duodenal mucosa	95% of B-NHLs, 5% of T
**33.**	F/66	**MCL**	PB	PB	PB	74% of B-NHLs, 24% of T, 2% of NK
**34.**	M/64	**MCL**	N	S	ingLN	94% of B-NHLs, 0.5% of B, 5.5% of T
**35.**	M/21	**BL**	N	FNAB	axLN	90% of B-NHLs, 3% of B, 6% of T, 1% of NK
**36.**	M/54	**BL**	N	FNAB	cLN	65% of B-NHLs, 2% of B, 23% of T, 6% of NK, 4% of M
**37.**	M/65	**EMZL**	E	FNAB	breastT	80% of B-NHLs, 18% of T, 2% of NK

Supplementary Table 1. Clinicopathological patients data of samples analysed by the optical tweezers. Abbreviation: F= female; M= male; UK=unknown; DLBCL, NOS= diffuse large B-cell lymphoma, not otherwise specified; GCB= germinal centre B-cell type; non-GCB= non-germinal centre B-cell type; FL= follicular lymphoma; HGBL= high grade B-cell lymphoma; MCL= mantle cell lymphoma; BL= Burkitt lymphoma; PCDLBCL= primary cutaneous diffuse large B-cell lymphoma; EMZL= extranodal marginal zone lymphoma; E= extranodal; N= nodal; FNAB= fine needle aspiration biopsy; S= surgically removed material; LN= lymph node; T= tumour; c= cervical; ax= axillary; ab= abdominal; ing= inguinal; st= soft tissue; PB= peripheral blood; FCM= flow cytometry analysis; B-NHLs= B-cell non-Hodgkin lymphoma cells; NK= natural killer lymphocytes; B= normal B-lymphocytes; T= normal T-lymphocytes; M= macrophages; D= debris.

**No.**	**Sex/Age**	**Diagnosis**	**Material**	**Site of involvement/ Source of surgical or FNAB specimen**
**1.**	F/82	Cholecystitis acute	S	common bile duct area
**2.**	F/76	Cholecystitis acute	S	common bile duct area
**3.**	F/65	Cholecystitis acute	S	common bile duct area
**4.**	F/80	Cholecystitis acute	S	common bile duct area
**5.**	M/65	Chronic colitis	S	abLN
**6.**	F/45	Chronic colitis	S	abLN
**7.**	M/29	Reactive lymphadenopathy	FNAB	cLN
**8.**	F/51	Reactive lymphadenopathy	FNAB	femoral LN
**9.**	F/58	Reactive lymphadenopathy	FNAB	cLN
**10.**	M/43	Reactive lymphadenopathy	S	cLN
**11.**	M/74	Reactive lymphadenopathy	FNAB	ingLN

**12.**	F/44	Reactive lymphadenopathy	S	cLN
**13.**	F/72	Reactive lymphadenopathy	S	axLN
**14.**	M/89	Reactive lymphadenopathy	FNAB	cLN
**15.**	F/44	Reactive lymphadenopathy	S	axLN

Supplementary Table 2. Clinicopathological controls data of samples analysed by the optical tweezers. Abbreviation: F= female; M= male; S= surgically removed material; FNAB= fine needle aspiration biopsy; LN= lymph node; c= cervical; ax= axillary; ab= abdominal; ing= inguinal.
